# Case report: Successful management of primary hyalinizing clear cell carcinoma in nasopharynx: a report of 2 cases and system analysis

**DOI:** 10.3389/fonc.2024.1384913

**Published:** 2024-05-31

**Authors:** Haiying Sun, Jie Yuan, Qing Cheng, Juanjuan Hu, Nana Han, Lisha Yi, Yun Zhu

**Affiliations:** ^1^ Department of Otorhinolaryngology, Union Hospital, Tongji Medical College, Huazhong University of Science and Technology, Wuhan, China; ^2^ Department of Stomatology, Tongji Hospital, Tongji Medical College, Huazhong University of Science and Technology, Wuhan, China; ^3^ Department of Anesthesiology, Union Hospital, Tongji Medical College, Huazhong University of Science and Technology, Wuhan, China

**Keywords:** hyalinizing clear cell carcinomas, nasopharynx, multimodal approach, recurrence-free, adjuvant chemoradiotherapy

## Abstract

Hyalinizing clear cell carcinomas (HCCCs) are infrequent, malignant tumors characterized by their low-grade nature. They typically originate from minor salivary glands. However, these tumors can potentially emerge in any location with minor salivary glands, including the nasopharynx. This report presents two cases of HCCC in females aged 61 and 72 years, with both tumors approximately 4 cm in size. In the first case, a 72-year-old female presented with recurrent bilateral epistaxis. Imaging studies revealed a nasopharyngeal mass, surgically excised, and histopathological analysis confirmed HCCC. Postoperatively, the patient received combined chemotherapy and radiotherapy, achieving a recurrence-free status 2.5 years later. The second case involves a 61-year-old female with a two-year history of bloody nasal discharge. Imaging studies identified a nasopharyngeal lesion, surgically removed, and histopathological examination confirmed HCCC. This patient underwent radiotherapy followed by combination chemotherapy with paclitaxel and carboplatin, displaying no signs of recurrence upon reevaluation after 10 months. These cases highlight the successful management of HCCC through a comprehensive, multimodal approach, integrating surgical intervention and adjuvant therapy. The favorable outcomes emphasize the significance of a thorough treatment strategy for HCCC in the nasopharynx, providing valuable insights for clinicians. Further studies are essential to enhance our understanding of this rare entity and refine treatment protocols for optimized patient outcomes.

## Introduction

Hyalinizing clear cell carcinoma (HCCC), also referred to as clear cell carcinoma (CCC) by the World Health Organization (WHO), is a rare tumor believed to originate from salivary glands, often presenting as an oral submucosal lesion in individuals of middle to advanced age (1). Although its primary occurrence is in the oral cavity, it can also uncommonly manifest in locations such as the trachea (2), bronchi (3) and nasopharynx (4). Initially described by Milchgrub et al. in 1994, this unique carcinoma exhibits distinctive histomorphology, characterized by infiltrating cords and nests of tumor cells featuring clear cytoplasm, all set within a hyalinized stroma (5).

In a case series conducted by Kauzman et al., nasopharyngeal HCCC exhibited a 1% prevalence among 98 reported cases of HCCC in English literature (6). HCCC displays a higher incidence in females and typically manifests as a small, indolent mass (7). The occurrence of local or distant metastases at the time of presentation is infrequent, with no instances (0 out of 10) of nasopharyngeal HCCC presenting with distant metastases reported (6, 8). Symptoms associated with nasopharyngeal HCCC include otorrhea, nasal congestion, epistaxis, and tinnitus (4, 7). Standard treatment approaches involve surgical resection and neck dissection in the presence of lymphadenopathy during the initial evaluation. Generally, the prognosis for HCCC is favorable, particularly when negative margins are achieved. In cases of positive surgical margins or notably aggressive tumors, radiotherapy is often employed to mitigate the risk of recurrence (7).

Our goal is to present detailed clinical presentations, diagnostic approaches, treatment modalities, and outcomes of two cases. Through these cases, we aim to underscore the importance of a comprehensive, multimodal treatment approach and provide valuable insights into the management of this rare entity in the nasopharynx.

## Case presentation

### Clinical history

The Ethics Committee of Tongji Medical College, Huazhong University of Science and Technology approved this study (IORG No: IORG0003571). This case report has been prepared in accordance with the CARE (Case Reports) guidelines, as available on the EQUATOR Network (https://www.equator-network.org/). Case 1: A 72-year-old female presented with a history of recurrent bilateral epistaxis for over a year, with a recent episode of significant bleeding occurring two days prior to seeking medical attention at Wuhan Union Hospital. The patient had been experiencing accompanying symptoms such as nasal congestion.

Case 2: A 61-year-old female sought medical attention due to a two-year history of bloody nasal discharge upon sniffing, which had progressively worsened. Additionally, the patient reported experiencing nasal congestion and ear fullness since April, prompting her visit to our hospital.

### Flexible nasopharyngoscopy

**Figure 1:**
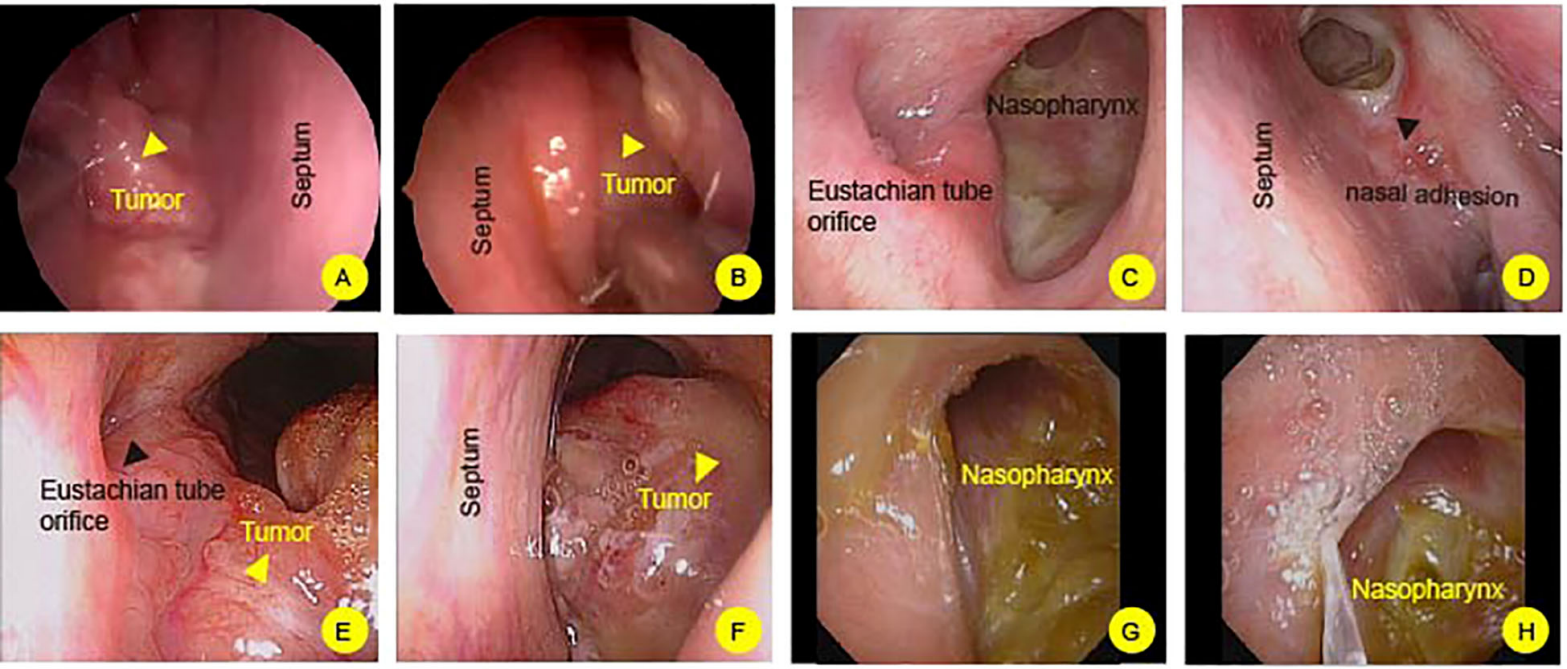
Flexible nasopharyngoscopy examination of nasopharynx. **(A, B)** In case 1, a prominent mass is observed on the nasopharynx (Yellow headarrow). The mass extends into posterior nasal cavity. The right nasal cavity shows postoperative adhesions (Black headarrow). **(C, D)** After 2.5 years of post-treatment follow-up, no tumor recurrence is observed in the nasopharyngeal region. **(E, F)** In Case 2, a mass is identified in the nasopharynx, characterized by an irregular surface (Yellow headarrow). **(G, H)** following an 8-month post-treatment surveillance, there is no evidence of tumor recurrence in the nasopharyngeal area.

For case 1, a large polypoid mass is observed on the right side of the nasopharynx, characterized by a smooth surface and approximately measuring 4*3 centimeters. It extends into the right posterior nasal cavity, and the orifices of the pharyngeal recess and pharyngeal orifice of the Eustachian tube cannot be visualized (**Figures 1A, B**). A 2.5-year follow-up post-treatment showed no evidence of tumor recurrence in the nasopharynx (**Figures 1C, D**). However, adhesive changes were noted in the right nasal cavity caused by radiation therapy (**Figure 1D**, Black arrowhead). Case 2, there is a narrowing of the upper, middle, and lower nasal meatus on the right side. A new growth is identified in the right-sided nasopharynx with an irregular surface. The orifices of the right pharyngeal recess and pharyngeal orifice of the Eustachian tube are not visualized. Additionally, the left pharyngeal recess shows signs of narrowing (**Figures 1E, F**). During the 8-year follow-up post-treatment, no recurrence of the nasopharyngeal tumor was observed (**Figures 1G, H**).

### Imaging

#### Magnetic resonance imaging

**Figure 2:**
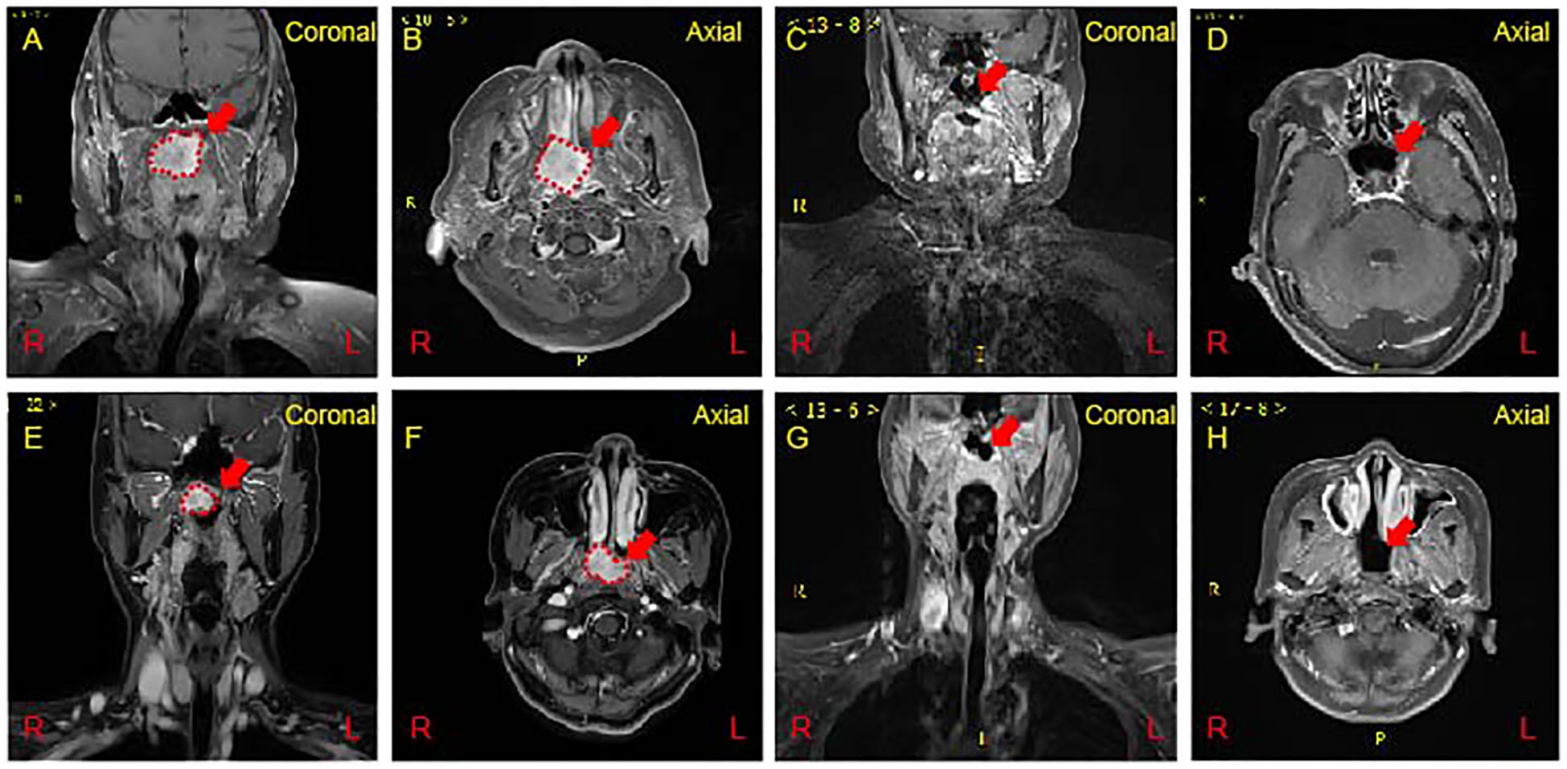
Magnetic Resonance Imaging (MRI) of the nasopharynx. **(A, B)** A polgs of Case 1 on MRI. A mass measuring approximately 38 * 32 mm was observed on the right side of the nasopharynx (Red dash line). Enhanced scanning reveals significant and uneven enhancement. **(C, D)** At 2.5 years post-treatment reveals the absence of any abnormal signal changes in the nasopharynx (Red arrow show). **(E, F)** An irregular mass measuring approximately 24 x 20 x 19 mm on the right side of the nasopharynx (Red dash line). Enhanced scan indicating significant and uneven enhancement of the nasopharyngeal mass. **(G, H)** Follow-up at 8 months post-treatment showing no abnormal signal changes in the nasopharynx (Red arrow show).

Case 1: A polypoid mass, measuring approximately 3.8 * 3.4 cm, was identified on the right lateral wall of the nasopharynx, exhibiting slightly prolonged T1 signal and longer T2 signal. The mass protrudes forward towards the right posterior nasal cavity, exerting downward compression or invasion into the right soft palate. It extends to involve the right pterygoid plate and the right lateral wall of the nasopharynx, partially invading the pharyngeal recess. Enhanced scanning demonstrates significant enhancement with relatively clear boundaries (**Figures 2A, B**). A 2.5-year follow-up post-treatment revealed no abnormal signal changes in the nasopharynx (**Figures 2C, D**).

Case 2: The right side of the nasopharynx showed noticeable thickening with an irregular T1-slightly prolonged T2 signal mass measuring approximately 24 * 20 * 19 mm. Enhanced scanning reveals significant and uneven enhancement. The right pharyngeal recess and the orifice of the pharyngotympanic tube are blocked, and the left pharyngeal recess becomes narrowed. The involvement extends to the right levator veli palatini and tensor veli palatini muscles. The slope and cranial base bone structures appear normal without evident abnormalities. Multiple small lymph nodes are noted in bilateral neck regions I-IV, with the right neck region II showing slightly larger lymph nodes, the largest measuring approximately 12 mm in short diameter. Enhanced scanning reveals uneven enhancement in the affected lymph nodes (**Figures 2E, F**). At an 8-month follow-up post-treatment, no abnormal signal changes were observed in the nasopharynx (**Figures 2G, H**).

### Pathological examination

**Figure 3:**
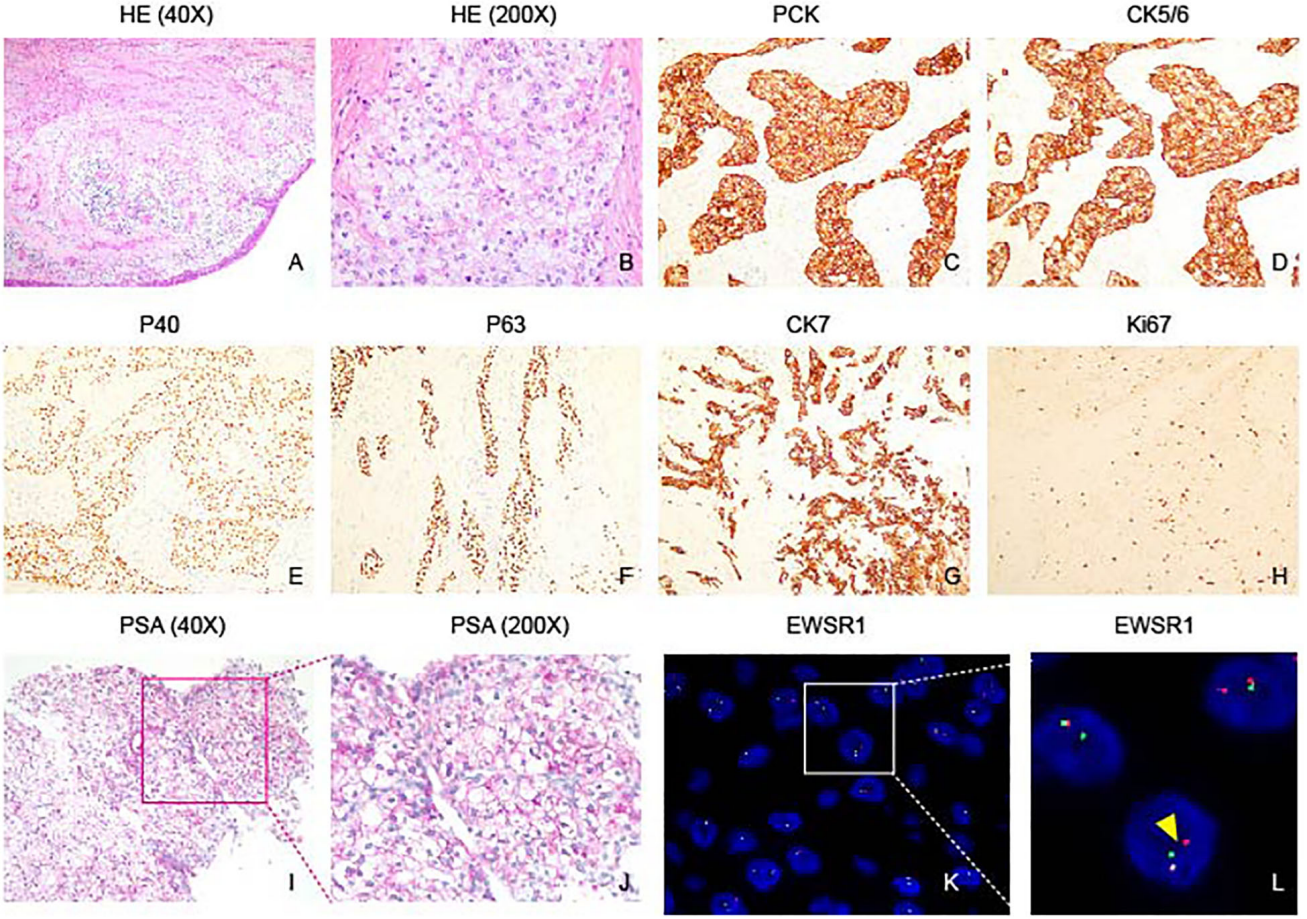
Histopathological features of nasopharyngeal hyalinizing clear cell carcinoma. **(A)** HE staining shows the tumor grows in a nest pattern and cord pattern. **(B)** Tumor cells characterized by rich cytoplasm, presenting as either transparent or lightly eosinophilic, and featuring round cell nuclei. **(C–G)** Immunohistochemical analysis demonstrates positive staining for PCK, CK5/6, P40, P63, and CK7 in the tumor. **(H)** Ki67 immunostaining reveals a low proliferation index of 5% in the tumor, indicating a relatively low rate of cell proliferation. **(I, J)** PAS staining highlights abundant glycogen within tumor cells. **(K, L)** Fluorescence *in situ* hybridization (FISH) shows spit signals, indicating EWSR1 rearrangement (Yellow arrowhead).

Hematoxylin and Eosin (HE) staining reveals an epithelial-like tumor with transparent and acidophilic cytoplasm (**Figures 3A, B**). The immunohistochemical profile of the tumor revealed positive staining for PCK, CK5/6, P40, P63, CK7 (**Figures 3C–H**), while being negative for CK20, S100, SMA, SOX10, PAX8, CAIX and CD10 (Data not shown). Additionally, Ki67 exhibited a low proliferation index (5%). Special staining with PAS was positive, suggesting glycogen-rich cytoplasm (**Figures 3I, J**). FISH analysis confirmed EWSR1 gene rearrangement (**Figures 3K, L**). Collectively, these findings are indicative of a diagnosis of clear cell carcinoma with a glassy cell variant. The positive expression of cytokeratins and the absence of neuroendocrine markers and other specific antigens support the epithelial origin of the tumor. The presence of EWSR1 gene rearrangement further contributes to the molecular characterization of the neoplasm. The combination of these immunohistochemical and molecular findings aids in accurate histopathological classification and has important implications for the diagnosis and potential management of the tumor.

### Treatment

The choice of treatment approach was based on the comprehensive evaluation of the patient’s condition, tumor characteristics, and consideration of the optimal therapeutic outcome (7, 9). The tumor was excised under nasal endoscopy, and histopathological examination of tissue samples from the anterior, posterior, left, right, upper, and lower margins revealed negative results. Postoperatively, the patient underwent six cycles of radiotherapy with a dose of 70 Gy in 33 fractions to the planning target volume for the primary tumor (DT PGTVp), and 60 Gy in 33 fractions to the planning clinical target volume (PCTV) with a simultaneous boost to the involved lymph nodes (v/2). Additionally, two cycles of paclitaxel and cisplatin sensitization were administered.

### Literature review

**Figure 4:**
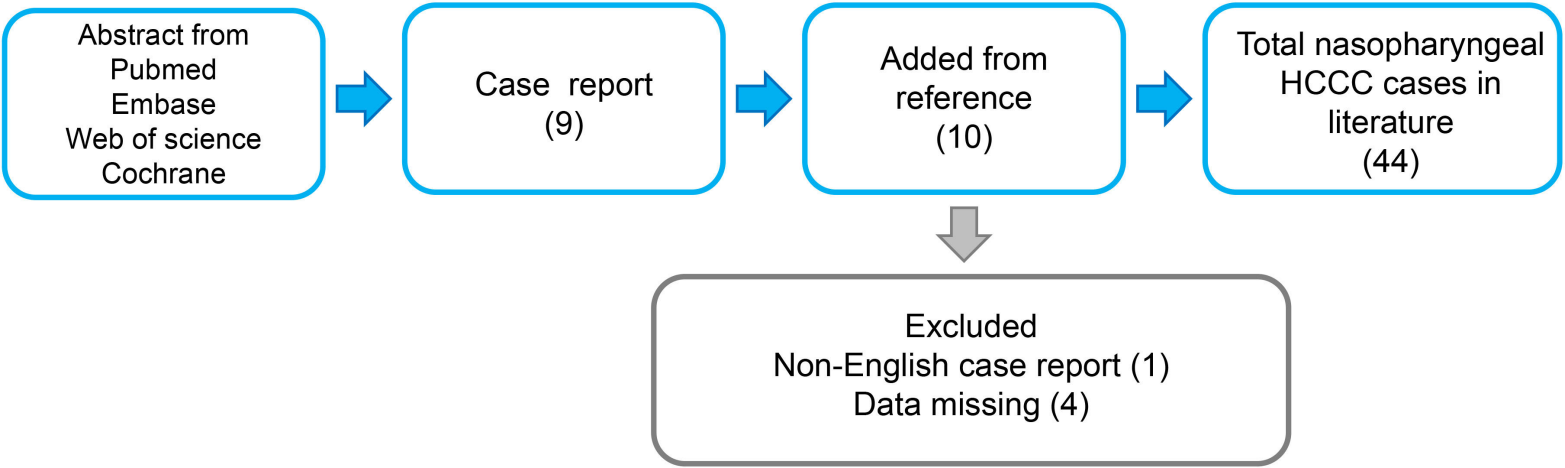
Schematic of literature review. HCCC, hyalinizing clear cell carcinoma.

A comprehensive review of 44 cases of Primary Nasopharyngeal Hyalinizing Clear Cell Carcinoma (HCCC) is presented (**Figure 4**). With the inclusion of the 2 cases reported in this study, a total of 46 cases were reviewed and analyzed. The information of these patients is summarized in **Table 1**. The investigation extends over an extensive duration, encompassing a heterogeneous array of age groups and symptomatic manifestations. The mean age at presentation was 52.61 years (range: 22-82), with an obvious female predominance (31 women and 15 men). Symptoms varied, with common presentations including nasal congestion, epistaxis, tinnitus, hearing loss, and respiratory issues. Treatment modalities employed were diverse and included surgery, radiotherapy, chemotherapy, and a combination of these approaches.

**Table 1:** Features of the primary nasopharyngeal hyalinizing clear cell carcinoma described in the present study.

Case	Study	Year	Age	Sex	Size(cm)	symptom	Treatment	Follow-up(month)
1	Tang (10)	1995	51	F	NA	Nasal congestion	S+RT	Recurrence 5 times, 144 mo
2	Cheng (11)	2008	63	F	3.99*3.42*4.85	repeated epistaxis,right-side tinnitus, and weight loss for 2 months	S+RT	NED, 12 mo
3	Antonescu (12)	2011	77	F	3.8	NA	S	NED, 12 mo
4	Sáenz (9)	2014	38	F	3.2*4.5*4.4	Nasal congestion, nasal voice quality change, and rhinorrhea for 5 months	S+RT+Chemo	NA
5	Albergotti (13)	2014	35	F	2.5	NA	S(debulking)+RT	DOD
6	Dosemane (14)	2015	22	F	2*2*1.5	Two episodes of minimal epistaxis and bilateral, progressive but partial nasal obstruction for 2 months	S+RT	NED, 36 mo
7	Nakano (8)	2015	27	F	2.5	Hearing loss and feeling of fullness in the right ear	S	NED, 18 mo
8	Nakashima (15)	2015	27	F	3.5*2.5	Right tinnitus and hearing disabilitly for 6 months	S	NED, 24 mo
9	Fukuda (16)	2015	63	F	1.4*1.4*1.7	An asymptomatic, slow growing, nasopharyngeal mass for 5 years	S	NED, 12 mo
10	Bishop (17)	2018	45	F	NA	NA	NA	NA
11			66	F	NA	NA	NA	NA
12			30	F	NA	NA	NA	NA
13			67	F	NA	NA	NA	NA
14	Zhao (18)	2018	62	M	1.8	NA	S	NED, 8 mo
15	Chapman (19)	2018	62	M	1	NA	S	NED, 5 mo
16	Malfitano (7)	2019	48	M	4.5*2.4*4	Nasal congestion, hearing loss	S+RT	NED, 2mo
17	Goyal (20)	2020	20	M	6.5*5.4*5.2	Epistaxis, proptosis, and difficulty in breathing for 1 year	S+RT	NED, 6mo
18	Arifi (21)	2022	63	M	NA	Headaches, left nasal obstruction, and repeated epistaxis for 6 months	RT+Chemo	SWT, 12 mo
19	Zhai (4)	2023	51	M	NA	NA	NA	NA
20			77	F	4.8	NA	S	DOD, 155 mo
21			62	F	3.2	NA	RT	DOD, 88 mo
22			76	F	NA	NA	NA	NA
23			63	M	5	NA	NA	NA
24			64	M	3.9	NA	RT	DOD, 50 mo
25			70	F	2.5	NA	RT	DOD, 50 mo
26			33	F	3	NA	S	NED, 61 mo
27			48	M	3.3	NA	S	NED, 57 mo
28			82	M	4.2	NA	S+RT	SWT, 58 mo
29			70	M	3	NA	S	NED, 34 mo
30			73	F	2.1	NA	S	NED, 29 mo
31			30	F	3	NA	S	NED, 281 mo
32			30	F	4.2	NA	S	NED, 36 mo
33			72	M	5.6	NA	S	DOD, 133 mo
34			64	F	2.5	NA	S	NED, 19 mo
35			39	M	2.3	NA	S	NED, 22 mo
36			32	F	2.4	NA	S	NED, 36 mo
37			69	F	2	NA	S	NED, 15 mo
38			31	M	2.5	NA	S	NED, 14 mo
39			34	M	4	NA	S+RT	NED, 6 mo
40			50	F	1.5	NA	S+RT	NED, 14 mo
41			33	F	4	NA	Chemo+S	SWT, 30 mo
42			71	F	4	NA	S+RT	SWT, 195 mo
43			59	F	3.1	NA	S	SWT, 6 mo
44			38	F	2.5	NA	S+RT	SWT, 192 mo
45	Present case		72	F	4	Recurrent bilateral epistaxis	S+Chemo+RT	NED, 30 mo
46	Present case		61	F	4	bloody nasal discharge of 2 years	S+RT	NED, 10 mo

chemo, chemotherapy; DOD, died of disease; F, female; M, male; NA, not applicable; NED, no evidence of disease; RT, radiotherapy; S, surgery; SWT, survival with tumo.

Follow-up periods ranged from a few months to several years (range: 2mo-155mo). Recurrence was observed in some cases, while others achieved No Evidence of Disease (NED) status. Unfortunately, there were instances of Disease-Related Deaths (DOD). The present study contributes valuable data to the understanding of Primary Nasopharyngeal HCCC, emphasizing the importance of tailored treatment approaches and the need for long-term follow-up to assess outcomes.

## Discussion

Hyalinizing clear cell carcinoma (HCCC) is a rare tumor with distinctive histomorphology, primarily associated with salivary glands but occasionally found in uncommon locations like the nasopharynx (6). In our presented cases, both patients exhibited nasopharyngeal HCCC, adding to the limited literature on this rare entity. The favorable outcomes observed in these cases, marked by the absence of recurrence at 2.5 years and 8 months postoperatively, underscore the successful management achieved through a comprehensive, multimodal approach.

The rarity of nasopharyngeal HCCC is highlighted by its prevalence of 1% in a case series, emphasizing the need for continued exploration of its clinical characteristics and optimal treatment strategies (7). The cases presented conform to the typical profile of HCCC, demonstrating positive immunohistochemical staining for cytokeratins, as well as focal GS positivity, consistent with glycogen-rich cytoplasm. The confirmation of EWSR1 gene rearrangement through FISH analysis further contributes to the diagnostic precision of this unique carcinoma.

The treatment strategy employed, consisting of surgical excision followed by adjuvant chemoradiotherapy, aligns with the standard approach for HCCC. Negative margins achieved during surgery are associated with a favorable prognosis, while the addition of adjuvant therapy serves to mitigate the risk of recurrence, especially in cases with positive surgical margins or aggressive tumor features.

Paclitaxel and cisplatin sensitization enhances the effectiveness of radiotherapy, while tailored radiotherapy doses ensure optimal tumor control while minimizing adverse effects on surrounding healthy tissue. This integrated approach highlights the importance of a personalized treatment strategy in managing HCCC effectively. The successful outcomes in our cases emphasize the significance of individualized and thorough treatment strategies for nasopharyngeal HCCC. However, given the limited number of reported cases, additional studies are imperative to refine treatment protocols and enhance our understanding of this rare carcinoma. Further investigations should focus on elucidating the molecular mechanisms underlying nasopharyngeal HCCC, which may aid in the development of targeted therapies and contribute to improved patient outcomes.

In conclusion, our report highlights the successful management of nasopharyngeal HCCC through a comprehensive, multimodal approach. The cases presented contribute valuable insights to the existing literature, emphasizing the need for continued research to refine treatment protocols and enhance our understanding of this rare carcinoma, ultimately optimizing outcomes for patients with nasopharyngeal HCCC.

## Data availability statement

The datasets presented in this study can be found in online repositories. The names of the repository/repositories and accession number(s) can be found below: no.

## Ethics statement

Written informed consent was obtained from the individual(s) for the publication of any potentially identifiable images or data included in this article.

## Author contributions

HS: Conceptualization, Funding acquisition, Writing – original draft. JY: Data curation, Methodology, Software, Writing – original draft. QC: Formal analysis, Investigation, Methodology, Writing – original draft. JH: Data curation, Methodology, Writing – original draft. NH: Investigation, Supervision, Visualization, Writing – review & editing. LY: Project administration, Supervision, Validation, Visualization, Writing – review & editing. YZ: Data curation, Formal analysis, Funding acquisition, Project administration, Supervision, Visualization, Writing – review & editing.
